# Education Research: Feasibility and Effect of Multinational Virtual Educational Series in Neonatal Neurology Across Latin America: The NeoNERd LatAm Initiative

**DOI:** 10.1212/NE9.0000000000200310

**Published:** 2026-05-01

**Authors:** Alexandra Santana Almansa, Gabriel Fernando Todeschi Variane, Manuel Vides-Rosales, Carlos Ivan Salazar Cerda, Alejandra Méndez-Fadol, Oscar DeLaGarza-Pineda, Carolina Serrano Tabares, Rumi Dasgupta, Juan Pablo Appendino

**Affiliations:** 1 Department of Neurology, Boston Children's Hospital, MA;; 2 Department of Neurology, University Pediatric Hospital, San Juan, PR;; 3 Division of Neonatology, Department of Pediatrics, Irmandade da Santa Casa de Misericórdia de São Paulo, Brazil;; 4 Clinical Research Department, Protecting Brains and Saving Futures, São Paulo, Brazil;; 5 Instituto de Neurociencias, Hospital de Diagnóstico, El Salvador;; 6 Department of Pediatrics, College of Medicine, University of Saskatchewan, Saskatoon, Canada;; 7 Pediatric Neurology Division, Department of Pediatrics, Jim Pattison Children's Hospital, Saskatoon, Saskatchewan, Canada;; 8 Departamento de Pediatría y Cirugía Infantil. Universidad de la Frontera, Temuco, Chile;; 9 Neurology Department, University Hospital “Dr. José E. González”–UANL, Monterrey, Mexico;; 10 Neurology Department, Clínica Universitaria Bolivariana, Medellín, Colombia; and; 11 Department of Pediatrics, Cumming School of Medicine, University of Calgary, Alberta, Canada.

## Abstract

**Background and Objectives:**

Neurologic disorders in the neonatal period are a leading cause of death and disability in low- and middle-income countries, including Latin America. Access to specialized neonatal neurocritical care remains limited, and educational opportunities are scarce. We aimed to evaluate the feasibility, reach, knowledge acquisition, and perceived value of a virtual educational series in neonatal neurology targeting health care professionals across Latin America.

**Methods:**

This prospective educational intervention included 6 case-based webinars delivered between October 2023 and April 2024 in Spanish via Zoom. The series, organized by the Newborn Brain Society's Latin American Task Force, was open-access and promoted through regional medical societies. Participants completed demographic surveys, pre- and postsession knowledge assessments, and standardized feedback forms. The virtual webinar series covered key neonatal neurologic topics, including hypoxic-ischemic encephalopathy, seizures, hypotonia, and neuromonitoring. Presenters were local trainees with expert moderator commentaries. Outcomes included webinar attendance, knowledge acquisition (multiple-choice questions), and satisfaction (5-point Likert scale). Responses were analyzed overall and for matched pre- and postsession participants.

**Results:**

A total of 1,424 participants from 24 countries attended the 6 webinars, with each session exceeding 100 attendees and 10 countries (meeting predefined feasibility criteria). Attendees were predominantly physicians (84%), although nurses, residents, and other professionals also participated; 23% attended multiple sessions. Knowledge scores improved significantly, with an 8%–18% increase in matched pre- vs postsession scores (*p* < 0.05). Participant satisfaction was high (mean feedback ∼4.8/5), and qualitative feedback highlighted the series' clinical relevance, interactivity, and value for practice.

**Discussion:**

This virtual educational initiative proved feasible, engaging, and effective in improving neonatal neurology knowledge across Latin America. Such tele-education programs may help reduce regional disparities in care.

## Introduction

Neurologic disorders in the newborn are a leading cause of death and disability in low- and middle-income countries (LMICs), including neonatal encephalopathy, complications of premature birth, and congenital anomalies.^1-3^ Despite a growing interest and motivated providers, neurologic care in Latin America Neonatal Intensive Care Units (NICUs) remains limited, including capability for therapeutic hypothermia and neuromonitoring. Care is not only limited but also highly variable, including variability in neurologic assessment and scoring tools, use of electroencephalography, use of therapeutic hypothermia, imaging, and outpatient follow-up.^4^

Previous studies have shown benefit of implementing tele-educational programs to decrease variability in care for patients with hypoxic ischemic encephalopathy treated with therapeutic hypothermia.^5,6^ Tele-education has the benefit of being able to reach providers world-wide in a cost effective manner, not only with the goal of improving services but also connecting providers and characterizing care in different regions.

The Newborn Brain Society, an international society that focuses on improving Neurologic Newborn Care, created the Latin American Task Force, with the goal of optimizing Neurologic NICU care in Latin America, promoting collaboration between providers, and providing educational opportunities. As part of this task force, we created an educational series titled NeoNERd LatAm (Neonatal Neurology Educational Rounds for Latin America). The goal of the educational series was to discuss high-yield topics, in a case-based manner, to improve awareness of common neurologic conditions in the newborn period and discuss how to optimize diagnostic evaluations and management. This study aimed to evaluate the feasibility and effectiveness of a virtual neonatal neurology educational series in Latin America, focusing on knowledge acquisition, user satisfaction, and knowledge gaps identification for future educational interventions. We hypothesized that it would be feasible to create a virtual educational series, attended broadly by providers from Latin America, improving knowledge regarding neonatal neurocritical care.

## Methodology

The NeoNERd LatAm Webinar Series consisted of monthly webinars, occurring the first Tuesday of every month from October 2023 to December 2023 and February 2024 to April 2024 via Zoom, with each session lasting 60–90 minutes. All webinars were held in Spanish. Webinars were advertised in the Newborn Brain Society website, and other pertinent medical societies across Latin America, including pediatrics, pediatric neurology, and neonatology medical societies. Registration was free of charge.

We defined feasibility as the successful implementation of all 6 planned webinars, with sustained audience engagement across the series. Predetermined feasibility thresholds included (1) achieving ≥100 attendees per session and (2) representation from ≥10 countries in each webinar. Learner engagement was defined using learner-reported engagement on postsession feedback surveys. We operationalized engagement as being achieved when ≥80% of respondents endorsed “agree” or “strongly agree” for 2 key items: *the session was interactive,* and *I would recommend this webinar to a colleague.* These thresholds were selected to capture both behavioral engagement (active participation) and perceived educational value (perceived interactivity). Repeat attendance was also measured among all attendees and among those answering the demographic survey (sustained attendance).

Webinars were organized and led by members of the Latin American Task Force. Each webinar was presented by a Neonatology or Child Neurology trainee from Latin America, and experts from Iberoamerica were invited both to supervise and aid in the discussion. High-yield webinar topics were decided based on expert opinion from both neurologist and neonatologist and members of the Latin American Task Force. Topics were selected based on expert consensus regarding high-priority subjects for providers caring for newborns in the NICU. These topics represent core knowledge that all clinicians involved in neonatal care, including neonatologists and child neurologists, should possess to optimize diagnosis and management. The curriculum was intentionally designed to cover complementary but distinct aspects of neonatal neurologic disorders. Topics included the following:

1. Diagnosis and treatment of hypoxic ischemic encephalopathy in newborns
2. Nonhypoxic ischemic encephalopathy
3. Neuromonitoring in neonates
4. Neonatal hypotonia
5. Neonatal seizures (acute symptomatic seizures, including those because of hypoxic ischemic injury, intracranial hemorrhage, infection, ischemic stroke, or metabolic disturbances)
6. Neonatal epilepsy (genetic epilepsies, brain malformations, and other structural or metabolic conditions).

Learning objectives were created for each webinar and approved by the Royal College of Physicians and Surgeons from Canada. CME credit was available for each webinar.

Webinars were case-based, with specific subtopics dedicated to each case. After the case presentation, a number of questions were discussed with the invited experts. To evaluate educational effectiveness, multiple-choice questions were added beginning with the second webinar and were administered immediately before and after each session, or each case. The same questions were used for both the pre- and postassessments, allowing for direct comparison of knowledge change. Educational effectiveness was defined a priori as a statistically significant increase in the percentage of correct responses from pre- to postassessment. Because matched (same-participant) comparisons provide a more accurate estimate of true learning, these were considered the primary analytic measure for determining effectiveness. Percent of correct answers were reported. These questions were developed collaboratively by the presenters and expert moderators and were designed to address the key learning objectives for each webinar. They assessed fundamental concepts essential for each topic, and clinically relevant points embedded within the case-based discussions, ensuring close alignment between the educational content and the assessment strategy.

For the first webinar, questions were administered only after the session, but we quickly realized that to optimize the evaluation of knowledge acquisition, it would be best to assess knowledge before and after the educational presentations. We also acquired demographic information and subjective feedback at the end of each session. Demographic information included medical role, specialty, and location of practice. Webinar feedback was acquired with Likert scale ratings to evaluate the perceived utility of the webinars (scale 1 to 5, being 1 = not useful at all and 5 = extremely useful). The same questions were asked after each webinar, including the following:

1. Were educational objectives met?
2. Did the webinar help you improve your knowledge on the topic?
3. Did it meet your expectations?
4. Did the webinar have information that is relevant to your practice?
5. Was the webinar interactive with the audience for 25% of the time?
6. Was it free of commercial influence?
7. Was the content relevant for your practice?
8. Were educational methods effective?
9. Would you recommend these educational webinars to a colleague?

We also asked open-ended questions about what was learned, what can be improved, and what other topics would be of interest. Chat was open throughout the session for questions and comments, and participants were able to control their audio and video; although, a moderator for each session was able to mute and turn-off cameras if appropriate. Statistical analysis was done using Microsoft Excel software (Version 16.66.1).

### Standard Protocol Approvals, Registrations, and Patient Consents

The study was deemed exempt from full Institutional Review Board review by the University of Calgary Research Ethics Board as there was no personal health information obtained, and participation in surveys and knowledge assessments was voluntary. The Board categorized the work as minimal-risk educational research/quality improvement and, therefore, exempt from further review.

### Data Availability

Deidentified data that support the findings of this study are available from the corresponding author on reasonable request.

## Results

**Figure 1:**
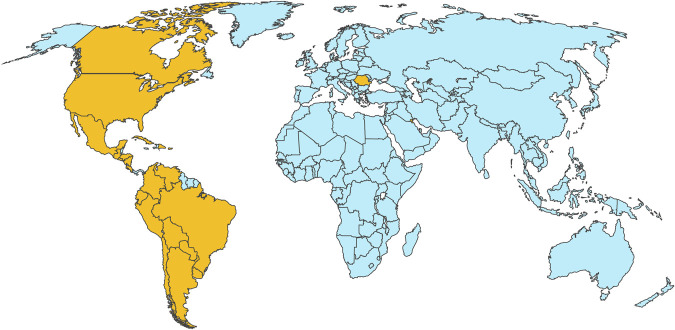
Geographic Distribution of Webinar Attendees (Yellow)

Total number of participants were 179, 192, 148, 343, 177, and 385, for webinars 1 through 6, respectively. Webinars lasted 60–90 minutes. Participants practiced in Argentina, Bolivia, Brazil, Canada, Chile, Colombia, Costa Rica, Cuba, Dominican Republic, Ecuador, El Salvador, Guatemala, Honduras, Kuwait, Mexico, Nicaragua, Paraguay, Peru, Portugal, Romania, Saint Lucia, United States, Uruguay, and Venezuela (Figure 1).

To evaluate multi-webinar participation among respondents, we examined repeated participants across all 6 demographic survey datasets. A total of 1,090 (76.5%) unique attendees and 334 (23.5%) repeat attendees were identified out of 1,424 of the total participants in the 6 webinars. From the unique attendees, 525 responded the demographic survey, 432 (82.3%) attended a single webinar, and 93 (17.7%) attended more than 1 session, with 30 individuals (32.3%) attending 3 or more webinars. Participants who completed the demographic questionnaire self-identified as physician (84%), resident (8%), nurses (6%), physical therapist (1%), and other (2%). About 73% practiced in the public setting and 27% practiced in the private setting.

All 6 planned webinars were successfully delivered as scheduled, meeting our predefined feasibility criteria. Each session surpassed the attendance threshold of 100 participants (range 148–385) and included attendees from at least 10 countries (range 10–17 countries per session). This confirms that the virtual series was feasible to implement and sustained broad regional engagement over 7 months.

Learner engagement metrics were high across all webinars. Attendance remained strong throughout the series (in fact peaking in later sessions), indicating sustained interest. Postsession survey responses also exceeded our engagement threshold: in every session, well over 80% of respondents agreed or strongly agreed that the webinar was interactive and that they would recommend it to a colleague. The average Likert rating for interactivity and recommend-to-colleague was 4.84 out of 5 (95% CI 4.81–4.86) across the series, indicating consistently strong engagement. Similarly, ≥80% of respondents agreed/strongly agreed in all other evaluation domains, such as perceived improvement in knowledge (mean 4.86, 95% CI 4.84–4.89), relevance to practice (4.87, 95% CI 4.84–4.90), meeting expectations (4.87, 95% CI 4.84–4.89), lack of commercial bias (4.89, 95% CI 4.85–4.93), and effectiveness of teaching methods (4.85, 95% CI 4.82–4.88).

**Figure 2:**
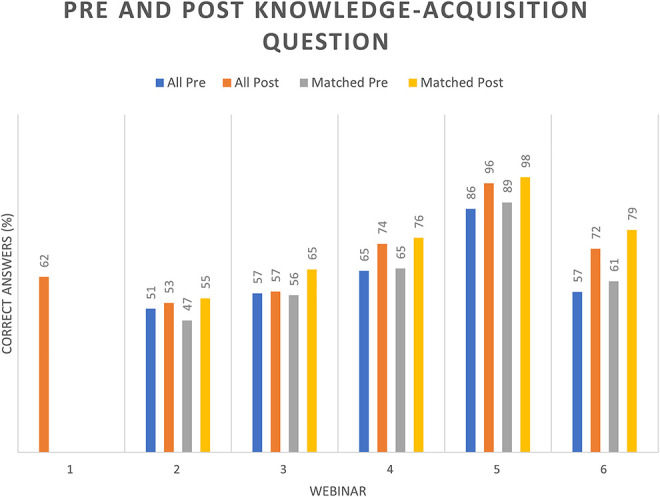
Percentage of Correct Answers per Webinar for all Participants and for Matched Participants

Across webinars 2–6, knowledge scores improved from an average of 63.8% (95% CI 55.9–71.7) to 71.7% (95% CI 64–79.2) postwebinar for all respondents and from 64.4% (95% CI 56.2–72.6) to 75.6% (95% CI 68.1–83.2) among matched respondents (*p* = 0.04, for matched improvement), as shown in Figure 2. The predefined criterion for educational effectiveness—a statistically significant improvement in matched pre- to postassessment scores—was achieved, as demonstrated by a significant increase among matched participants. These results indicate that the series effectively enhanced participant knowledge across key neonatal neurology topics.

**Figure 3:**
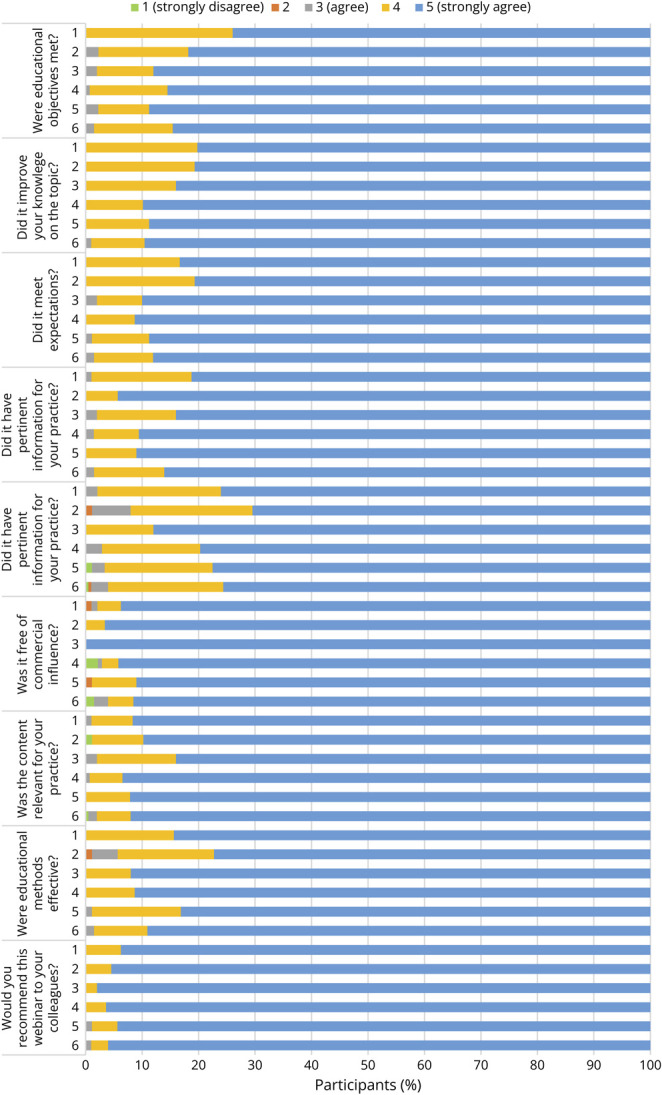
Subjective Feedback Ratings per Webinar

Webinar 1 included only postsession assessment (10 questions), with correct responses ranging from 10% to 88% (mean 62.3%, 95% CI 43.1–81.6). After instituting pre- and postquestionnaires from webinar 2 onward, we observed knowledge gains for each session. Table 1 summarizes the knowledge changes across all questions for webinars 2 to 6 comparing pre- and post-testing in each question for both, overall and matched responders. Notice the significant “*p*” values (*p* < 0.05) for the paired *t* test in matched responders for webinars 3–6, confirming the educational effectiveness of our intervention. Table 1 also presents the overall feedback rating for each webinar, and Figure 3 provides a detailed breakdown of feedback ratings across sessions.

**Table 1:** Knowledge Scores and Learner Feedback Across NeoNERd LatAm Webinars

Webinar	Total participants (n)	Countries represented	Knowledge assessment format	Knowledge scores/Change (all participants)	Matched-participant change	*p* Value	Average Likert rating (1–5)
1	179	12	Postwebinar only (10 questions)	Correct responses ranged 10%–88% (mean 62%)	Not applicable	—	4.83 (95% CI 4.86–4.81)
2	192	14	5 pre/post questions	+16%, −21%, +8%, +13%, −6%	+30%, −4%, −4%, +17%, 0%	0.77 (all); 0.32 (matched)	4.84 (95% CI 4.87–4.80)
3	148	10	5 pre/post questions	+1%, +12%, +1%, −5%, −7%	+17%, +17%, +4%, +8%, 0%	0.87 (all); 0.05 (matched)	4.89 (95% CI 4.92–4.86)
4	343	17	6 pre/post questions	+4%, +7%, −8%, +16%, +18%, +20%	+3%, +10%, −5%, +22%, +16%, +18%	0.08 (all); 0.04 (matched)	4.89 (95% CI 4.91–4.87)
5	177	15	6 pre/post questions	+4%, +7%, +14%, +23%, +7%, 0%	+2%, +12%, +9%, +24%, +4%, +2%	0.04 (all); 0.05 (matched)	4.87 (95% CI 4.90–4.84)
6	385	17	6 pre/post questions	+25%, +16%, +6%, +21%, +20%, +4%	+32%, +13%, +7%, +23%, +19%, +14%	0.007 (all); 0.004 (matched)	4.85 (95% CI 4.87–4.83)

Qualitative feedback from open-ended survey questions was overwhelmingly positive. Participants consistently described the series as highly valuable, clinically relevant, and even practice-changing. Many respondents reported improved skills or understanding in areas such as recognizing neonatal seizure semiology, differentiating central vs peripheral hypotonia, interpreting amplitude-integrated EEG and EEG, and distinguishing hypoxic-ischemic from nonhypoxic encephalopathy. They highlighted that structured algorithms, case discussions, and real patient videos/EEG tracings helped strengthen diagnostic reasoning and informed concrete management decisions. Examples of reported changes in practice included earlier and more appropriate use of antiseizure medications, refined criteria for initiating therapeutic hypothermia, and better indications for genetic/metabolic workups. A number of participants noted that the content equipped them to advocate for neuromonitoring and other needed resources in their local units. The interactive, case-based format—featuring audience polls, panel discussions, and opportunities for Q&A—was frequently praised as engaging and effective. Suggestions for improvement were relatively minor and focused on logistics (e.g., allowing more time for audience questions and poll discussion, minor technical fixes, providing subtitles or handouts, and adjusting session times to accommodate different time zones). These comments suggest a high overall satisfaction with the educational design and content (Table 2 provides example quotes).

**Table 2:** Summary of Qualitative Feedback Across Webinars

Domain	Summary of participant feedback	Representative themes
Overall Perceived Value	Webinars were consistently described as highly valuable, clinically relevant, and directly applicable to practice	*“Excelente”* (Excellent)*, “Muy útil”* (Very helpful)*, “Práctico”* (Very practical)*, “Inmejorable”* (Can't be improved)*, “Impactará mi práctica diaria”* (Will have a great impact on my everyday practice)
Knowledge & Skills Gained	Participants reported improved ability to diagnose and manage common neonatal neurologic conditions	• Recognition of neonatal seizure semiology and EEG/aEEG patterns • Differentiation of central vs peripheral hypotonia • Improved classification of encephalopathies • Understanding etiologies of neonatal epilepsy and metabolic disorders
Clinical Practice Impact	Learners adopted or planned to adopt updated management strategies and more structured diagnostic approaches	• Rational use of antiseizure medications and when to discontinue them • Earlier activation of therapeutic hypothermia pathways • More appropriate ordering of genetic/metabolic testing • Clearer neurologic examinations and triage
Usefulness of Educational Strategies	Case-based, interactive design was highly valued across all sessions	• Real EEG/aEEG tracings and neuroimaging • Video-based physical exams • Clinical algorithms • Polls and live Q&A • Multidisciplinary expert panel
Systems-Level Influence	Webinars supported advocacy for improved neonatal neurology infrastructure, especially in low-resource settings	• Need for aEEG/continuous EEG • Standardized seizure pathways • Access to genetic testing • Implementation of neonatal neurologic protocols
Suggestions for Improvement	Feedback focused mainly on logistics rather than content quality	• More time for Q&A and poll responses • Larger/clearer display of EEG and imaging • Improved connection stability • Subtitles or bilingual options • Presession reading materials or PDFs • Slightly shorter or earlier sessions for international attendees
Overall Satisfaction	High satisfaction across all webinars, with strong requests for more sessions and expanded topics	*“Sigan así”* (Continue like this), *“Muy interactivas”* (Very interactive), *“Perfectas”* (Perfect), *“Deseamos más sesiones”* (We want more sessions), *“Excelente metodología”* (Excellent methodology)

## Discussion

Creating and organizing an international, virtual, case-based educational webinar series across Latin America to improve knowledge of neonatal neurocritical care was feasible, with excellent feedback, robust participation, and improvement of knowledge. Feasibility was further supported by surpassing predefined attendance and geographic-reach thresholds in every session, confirming that the structure and delivery model were practical and scalable for the intended audience. High learner engagement was demonstrated through sustained participation across all webinars and consistently strong subjective ratings, with ≥80% of respondents reporting the sessions to be interactive and recommendable. These findings confirm that the series not only reached a broad audience but also maintained active involvement throughout the educational intervention. The series also met our predefined measure of educational effectiveness. Knowledge acquisition improved across all webinars in which pre- and post-testing was implemented, with a statistically significant increase in matched participant scores. This suggests that the case-based, interactive format supported measurable short-term learning gains.

To maximize interaction and practical education, webinars were case-based, typically including 3 cases per webinar, with discussion after topic. Cases were drawn from the clinical experiences of patients treated at local institutions, including medical history, diagnostic studies, and limitations in care based on local resources. We were able to foster discussion across providers from Latin America, focused on how to optimize care with a range of available resources. Although the moderator, presenters, and experts primarily guided the discussion, there was ample audience participation, fostering discussion and collaboration between providers across Latin America. Throughout the discussion, a recurrent theme in all webinars was the variability in practice across Latin America, even within the same country. This discussion prompted the need for advocacy efforts to improve access to resources and services in Latin America. It also emphasized the importance of multicountry educational sessions to expand knowledge, how to advocate for our patients, and how to improve care. Further details about the variability of services will be reported on a different manuscript as it is not the aim of this work.

Attendees practiced in 24 countries, including lower-middle, upper-middle, and high-income countries.^7^ A total of 1,424 participants attended. Most participants were practicing physicians, but residents, physical therapists, and nurses also attended. Around three-fourths practiced in public health care. We did not perform subgroup analyses (e.g., by medical role, specialty, or practice location) because of the highly uneven distribution of participants across categories. Because more than four-fifths of attendees were practicing physicians, the very small sample sizes in other subgroups (residents, nurses, and physical therapists) would have resulted in underpowered and potentially misleading comparisons. Future iterations of the program, with more balanced representation of professional groups, may allow for more nuanced analyses of engagement and knowledge acquisition across learner types. For future sessions, we aim to increase attendance of residents, physical therapists, and nurses, which could potentially be achieved by offering webinars led by nurses or physical therapists in addition to physicians. This effort fostered equity within neonatal neurology, given the wide reach within Latin America, use of primary language (Spanish), and free-of-charge access. A study by Culquichicón et al.^8^ showed that compared with other countries, Latin America has a relatively low number of online courses, which limits accessible education. Therefore, it is crucial to continue expanding tele-education initiatives across Latin America to foster equitable access to education and care.

Knowledge acquisition was assessed through pre- and postsession multiple-choice questions implemented beginning with the second webinar, allowing evaluation of changes in participant performance. Overall, the average improvement in knowledge per webinar ranged from 1% to 15% for all participants and from 8% to 18% for matched participants, which was statistically significant. Gains were higher for most webinars when responses were matched by participant, supporting that matched analyses provide a more accurate estimate of learning effects and may represent meaningful gains for brief online educational interventions. Previous studies of webinar-based medical education have similarly reported improvements in learner knowledge and high perceived educational value after short virtual sessions.^9-11^ Short-term improvements in knowledge are widely considered an important early step toward strengthening clinical reasoning and supporting more consistent decision making in complex care environments. Qualitative feedback from participants further suggested that these gains translated into greater confidence in key clinical tasks, including recognition of neonatal seizures, interpretation of neuromonitoring, and application of diagnostic algorithms.

Although the present study did not directly evaluate changes in clinical practice, previous tele-education initiatives have demonstrated the potential to influence care delivery. Collaborative educational networks have been associated with reduced variability in neonatal care and improved adherence to evidence-based practices, such as the use of therapeutic hypothermia for neonatal encephalopathy.^5,12^ In this context, scalable virtual educational networks may play an important role in strengthening clinical capacity and reducing disparities in neonatal neurologic care across resource-variable settings.

Regarding feedback, we repeated the same survey after each webinar to evaluate if educational objectives were met, if it led to perceived improvement of knowledge on the topic, if it had pertinent information for clinical practice, if it was free of commercial influence, and if educational methods were effective. We received exceptional feedback, with an outstanding majority rating that webinars met educational objectives and were relevant to their clinical practice. We also had open answer questions, with highly positive feedback. We asked the audience what topics they were interested in learning more about, which will inform and be based on our educational series in the next academic cycle.

Previous studies have shown similar benefits of international, free, webinar educational series. D'Onofrio et al. reported their experience with a free epilepsy educational webinar series through the Canadian League Against Epilepsy with international attendance and high satisfaction. For this series, monthly webinars were done from March 2021 to September 2023, with a median attendance of 118 per session.^9^ Similarly, Depypere et al. showed that a series of 24 educational webinars done by The European Society of Thoracic Surgeons, reaching 76 countries, received excellent feedback with improvement in clinical knowledge.^10^ It has been shown that webinars improve access to educational material, related to both physical accessibility, and reduced time constraints and cost.^11^ Furthermore, educational webinars can lead to improved clinician-reported patient experience and outcomes.^12^ Leandro et al. showed that implementation of a videoconference series consisting of 12 biweekly webinars led to decreased variability in care for neonates with hypoxic-ischemic encephalopathy treated with therapeutic hypothermia.^5^

Limitations of this study included change in the methods of knowledge assessment after the first webinar and challenges with polling on a virtual platform. The use of self-reported feedback and having limited outcome measures (knowledge improvement only) are inherent limitations. Furthermore, the lack of long-term follow-up precludes understanding the effect on clinical decision making or patient outcomes. Although we had attendants from most countries in Latin America, we did not have participants from Panama. In addition, we did not collect data on participants' sex, ethnicity, or training location. This lack of demographic information limits our ability to assess whether the program reached a representative audience or if differences in engagement and learning existed across subgroups. Future iterations should capture these demographics to ensure inclusive reach and to identify any such differences in effect.

In the future, we will launch a second round of the NeoNERd LatAm educational webinar series, focused on topics proposed by the audience during the first webinar series to address local needs. We will continue to assess learning through knowledge-acquisition questions (pre- and postsession questions) and aim to evaluate effects on clinical practice. We hope to expand our reach to all countries of Latin America and beyond and broaden our audience to other neonatal care providers (including nurses, physiotherapists, lactation specialists, and others) to create an inclusive educational network environment.

To ensure the long-term sustainability of the NeoNERd LatAm initiative, ongoing support and engagement are vital. The program's volunteer-driven, low-cost model facilitated its initial success; however, maintaining momentum may benefit from formal backing by medical societies or sponsors to provide resources for coordination, technology, and expansion. Our virtual, case-based model is inherently scalable and can be adapted to other low- and middle-income regions by collaborating with local champions, delivering content in the region's primary language (Spanish), and tailoring topics to the local context. For example, similar neonatal neurology series could be conducted in other regions if content is provided in relevant languages and cultural context (e.g., Portuguese for Brazil or French for francophone Africa), ensuring that examples and discussions resonate with regional practice. Crucially, keeping such programs free-of-charge is important to maximize participation in resource-limited settings, as demonstrated by our experience.

International professional societies can further support inclusivity by helping remove barriers to participation. We recommend that global neurology and neonatal organizations consider waiving or subsidizing fees for attendees from resource-limited settings, offering hybrid access (in-person and virtual) to conferences and educational events, and providing translation or multilingual materials during webinars. Measures such as these—waived registration costs, virtual attendance options, and language translation—would encourage broader global engagement. The success of NeoNERd LatAm underscores that eliminating cost and language barriers enables wide participation; if international societies adopt similar strategies, it could greatly enhance equity and inclusivity in specialized medical education worldwide.

In conclusion, our work demonstrates that virtual education in neuro-neonatology is both feasible and effective in enhancing knowledge acquisition in Latin America, with the potential to positively influence patient care and outcomes. The high level of engagement and positive reception of our seminars highlight the strong interest in this educational approach, motivating us to further expand its reach, including other health care providers on the team. Future research should focus on assessing the long-term effect of these educational initiatives on clinical practices and patient outcomes.

## Glossary

NICU: Neonatal Intensive Care Unit
